# Supplementary material for: Modeling glioblastoma heterogeneity as a dynamic network of cell states
Source: Mol Syst Biol. 2021 Sep 16;17(9):e10105. doi: 10.15252/msb.202010105 (PMC8444284; doi:10.15252/msb.202010105)
Supplement: Supplementary file 6 — Source Data for Figure 5 [file MSB-17-e10105-s004.zip › Figure5A_sourcedata/GSEA_3017/nosigdb_stateB.GseaPreranked.1622795021725/RICHARDS_NOWAKOWSKI_SCIENCE_2017_MGE_DIV_UPREG.html]

Details for gene set RICHARDS\_NOWAKOWSKI\_SCIENCE\_2017\_MGE\_DIV\_UPREG[GSEA]

|  || Dataset | state43017 |
| Phenotype | NoPhenotypeAvailable |
| Upregulated in class | na\_pos |
| GeneSet | RICHARDS\_NOWAKOWSKI\_SCIENCE\_2017\_MGE\_DIV\_UPREG |
| Enrichment Score (ES) | 0.5390836 |
| Normalized Enrichment Score (NES) | 3.465573 |
| Nominal p-value | 0.0 |
| FDR q-value | 0.0 |
| FWER p-Value | 0.0 |
Table: GSEA Results Summary

  

Fig 1: Enrichment plot: RICHARDS\_NOWAKOWSKI\_SCIENCE\_2017\_MGE\_DIV\_UPREG      
 Profile of the Running ES Score & Positions of GeneSet Members on the Rank Ordered List

  

| PROBE | GENE SYMBOL | GENE\_TITLE | RANK IN GENE LIST | RANK METRIC SCORE | RUNNING ES | CORE ENRICHMENT || 1 | HIST1H4C |  |  | 0 | 1.373 | 0.0120 | Yes |
| 2 | HIST1H1B |  |  | 1 | 1.272 | 0.0231 | Yes |
| 3 | HIST1H1D |  |  | 2 | 1.180 | 0.0335 | Yes |
| 4 | TOP2A |  |  | 3 | 1.079 | 0.0429 | Yes |
| 5 | HIST1H1C |  |  | 4 | 1.041 | 0.0520 | Yes |
| 6 | HIST1H1E |  |  | 5 | 0.983 | 0.0606 | Yes |
| 7 | RRM2 |  |  | 7 | 0.875 | 0.0663 | Yes |
| 8 | FAM111A |  |  | 10 | 0.813 | 0.0695 | Yes |
| 9 | UBE2C |  |  | 11 | 0.802 | 0.0765 | Yes |
| 10 | TPX2 |  |  | 12 | 0.801 | 0.0835 | Yes |
| 11 | NUSAP1 |  |  | 13 | 0.801 | 0.0906 | Yes |
| 12 | HMGB2 |  |  | 14 | 0.786 | 0.0974 | Yes |
| 13 | FBXO5 |  |  | 15 | 0.785 | 0.1043 | Yes |
| 14 | NCAPG |  |  | 16 | 0.778 | 0.1111 | Yes |
| 15 | SPC25 |  |  | 17 | 0.763 | 0.1178 | Yes |
| 16 | PRC1 |  |  | 18 | 0.756 | 0.1244 | Yes |
| 17 | CDCA2 |  |  | 19 | 0.751 | 0.1310 | Yes |
| 18 | ESCO2 |  |  | 20 | 0.742 | 0.1375 | Yes |
| 19 | GTSE1 |  |  | 22 | 0.732 | 0.1419 | Yes |
| 20 | HJURP |  |  | 23 | 0.731 | 0.1483 | Yes |
| 21 | KIF23 |  |  | 24 | 0.721 | 0.1546 | Yes |
| 22 | ASPM |  |  | 26 | 0.695 | 0.1587 | Yes |
| 23 | NDC80 |  |  | 27 | 0.695 | 0.1648 | Yes |
| 24 | ATAD2 |  |  | 28 | 0.685 | 0.1708 | Yes |
| 25 | KIF15 |  |  | 29 | 0.684 | 0.1768 | Yes |
| 26 | ARHGAP11A |  |  | 30 | 0.683 | 0.1828 | Yes |
| 27 | CENPF |  |  | 34 | 0.670 | 0.1828 | Yes |
| 28 | KIF4A |  |  | 37 | 0.662 | 0.1846 | Yes |
| 29 | ANLN |  |  | 39 | 0.659 | 0.1885 | Yes |
| 30 | RAD51AP1 |  |  | 40 | 0.657 | 0.1942 | Yes |
| 31 | SMC4 |  |  | 41 | 0.655 | 0.1999 | Yes |
| 32 | MELK |  |  | 42 | 0.655 | 0.2057 | Yes |
| 33 | CENPE |  |  | 43 | 0.654 | 0.2114 | Yes |
| 34 | NUF2 |  |  | 44 | 0.651 | 0.2171 | Yes |
| 35 | PRR11 |  |  | 45 | 0.649 | 0.2228 | Yes |
| 36 | KIF18A |  |  | 48 | 0.639 | 0.2244 | Yes |
| 37 | DHFR |  |  | 49 | 0.638 | 0.2300 | Yes |
| 38 | NCAPG2 |  |  | 50 | 0.636 | 0.2356 | Yes |
| 39 | CDCA5 |  |  | 52 | 0.632 | 0.2392 | Yes |
| 40 | CKAP2 |  |  | 53 | 0.628 | 0.2447 | Yes |
| 41 | FANCI |  |  | 56 | 0.615 | 0.2461 | Yes |
| 42 | MYBL2 |  |  | 58 | 0.612 | 0.2495 | Yes |
| 43 | CKAP2L |  |  | 59 | 0.611 | 0.2549 | Yes |
| 44 | CENPU |  |  | 60 | 0.605 | 0.2602 | Yes |
| 45 | CENPK |  |  | 62 | 0.603 | 0.2635 | Yes |
| 46 | TYMS |  |  | 63 | 0.601 | 0.2687 | Yes |
| 47 | BUB1B |  |  | 64 | 0.596 | 0.2740 | Yes |
| 48 | CCDC34 |  |  | 65 | 0.595 | 0.2792 | Yes |
| 49 | KIF2C |  |  | 66 | 0.591 | 0.2843 | Yes |
| 50 | SMC2 |  |  | 68 | 0.586 | 0.2875 | Yes |
| 51 | CDK1 |  |  | 69 | 0.585 | 0.2926 | Yes |
| 52 | H2AFX |  |  | 71 | 0.576 | 0.2957 | Yes |
| 53 | CKS1B |  |  | 72 | 0.575 | 0.3007 | Yes |
| 54 | RACGAP1 |  |  | 75 | 0.572 | 0.3018 | Yes |
| 55 | FOXM1 |  |  | 76 | 0.570 | 0.3068 | Yes |
| 56 | MAD2L1 |  |  | 77 | 0.569 | 0.3118 | Yes |
| 57 | DBF4 |  |  | 79 | 0.567 | 0.3148 | Yes |
| 58 | CDCA8 |  |  | 82 | 0.562 | 0.3158 | Yes |
| 59 | CKS2 |  |  | 83 | 0.558 | 0.3207 | Yes |
| 60 | KIF14 |  |  | 85 | 0.557 | 0.3236 | Yes |
| 61 | E2F7 |  |  | 86 | 0.556 | 0.3285 | Yes |
| 62 | AURKB |  |  | 89 | 0.548 | 0.3293 | Yes |
| 63 | SPC24 |  |  | 91 | 0.544 | 0.3321 | Yes |
| 64 | CENPM |  |  | 92 | 0.544 | 0.3369 | Yes |
| 65 | CCNB1 |  |  | 93 | 0.544 | 0.3417 | Yes |
| 66 | NCAPH |  |  | 94 | 0.543 | 0.3464 | Yes |
| 67 | PBK |  |  | 95 | 0.540 | 0.3511 | Yes |
| 68 | XRCC2 |  |  | 98 | 0.530 | 0.3519 | Yes |
| 69 | SKA3 |  |  | 99 | 0.525 | 0.3565 | Yes |
| 70 | TMEM106C |  |  | 100 | 0.523 | 0.3610 | Yes |
| 71 | RRM1 |  |  | 103 | 0.520 | 0.3617 | Yes |
| 72 | CMC2 |  |  | 105 | 0.516 | 0.3642 | Yes |
| 73 | CCNF |  |  | 106 | 0.513 | 0.3687 | Yes |
| 74 | CEP135 |  |  | 109 | 0.513 | 0.3693 | Yes |
| 75 | UBE2T |  |  | 110 | 0.512 | 0.3738 | Yes |
| 76 | CDCA3 |  |  | 111 | 0.511 | 0.3782 | Yes |
| 77 | DIAPH3 |  |  | 112 | 0.510 | 0.3827 | Yes |
| 78 | TUBB4B |  |  | 114 | 0.507 | 0.3852 | Yes |
| 79 | RAD21 |  |  | 115 | 0.506 | 0.3896 | Yes |
| 80 | BIRC5 |  |  | 116 | 0.504 | 0.3940 | Yes |
| 81 | CLSPN |  |  | 117 | 0.503 | 0.3984 | Yes |
| 82 | CEP152 |  |  | 119 | 0.502 | 0.4008 | Yes |
| 83 | ASF1B |  |  | 120 | 0.501 | 0.4052 | Yes |
| 84 | DUT |  |  | 124 | 0.494 | 0.4037 | Yes |
| 85 | E2F8 |  |  | 125 | 0.493 | 0.4080 | Yes |
| 86 | TMPO |  |  | 127 | 0.492 | 0.4103 | Yes |
| 87 | ITGB3BP |  |  | 128 | 0.490 | 0.4146 | Yes |
| 88 | LMNB2 |  |  | 129 | 0.488 | 0.4189 | Yes |
| 89 | SKA1 |  |  | 131 | 0.487 | 0.4212 | Yes |
| 90 | SHCBP1 |  |  | 133 | 0.484 | 0.4235 | Yes |
| 91 | GMNN |  |  | 135 | 0.484 | 0.4258 | Yes |
| 92 | NCAPD2 |  |  | 136 | 0.483 | 0.4300 | Yes |
| 93 | TTK |  |  | 137 | 0.482 | 0.4342 | Yes |
| 94 | CENPL |  |  | 139 | 0.478 | 0.4364 | Yes |
| 95 | C21orf58 |  |  | 142 | 0.470 | 0.4366 | Yes |
| 96 | FANCD2 |  |  | 149 | 0.461 | 0.4289 | Yes |
| 97 | ORC6 |  |  | 150 | 0.461 | 0.4329 | Yes |
| 98 | KIF22 |  |  | 152 | 0.459 | 0.4350 | Yes |
| 99 | NCAPD3 |  |  | 153 | 0.458 | 0.4390 | Yes |
| 100 | CDC45 |  |  | 154 | 0.456 | 0.4430 | Yes |
| 101 | SPAG5 |  |  | 156 | 0.455 | 0.4450 | Yes |
| 102 | MCM8 |  |  | 158 | 0.454 | 0.4471 | Yes |
| 103 | POLQ |  |  | 162 | 0.450 | 0.4451 | Yes |
| 104 | CENPQ |  |  | 163 | 0.446 | 0.4490 | Yes |
| 105 | TK1 |  |  | 167 | 0.444 | 0.4470 | Yes |
| 106 | BRIP1 |  |  | 168 | 0.443 | 0.4509 | Yes |
| 107 | ATAD5 |  |  | 170 | 0.442 | 0.4528 | Yes |
| 108 | BRCA1 |  |  | 171 | 0.440 | 0.4567 | Yes |
| 109 | CDK5RAP2 |  |  | 173 | 0.438 | 0.4585 | Yes |
| 110 | BRCA2 |  |  | 174 | 0.436 | 0.4624 | Yes |
| 111 | RBBP8 |  |  | 176 | 0.436 | 0.4642 | Yes |
| 112 | DEPDC1B |  |  | 177 | 0.436 | 0.4680 | Yes |
| 113 | MKI67 |  |  | 179 | 0.436 | 0.4699 | Yes |
| 114 | FEN1 |  |  | 180 | 0.435 | 0.4737 | Yes |
| 115 | USP1 |  |  | 185 | 0.430 | 0.4696 | Yes |
| 116 | MIS18BP1 |  |  | 186 | 0.430 | 0.4734 | Yes |
| 117 | DDIAS |  |  | 187 | 0.428 | 0.4771 | Yes |
| 118 | MGME1 |  |  | 188 | 0.428 | 0.4809 | Yes |
| 119 | BARD1 |  |  | 189 | 0.427 | 0.4846 | Yes |
| 120 | CKAP5 |  |  | 192 | 0.426 | 0.4844 | Yes |
| 121 | MND1 |  |  | 193 | 0.424 | 0.4881 | Yes |
| 122 | NEDD1 |  |  | 195 | 0.423 | 0.4899 | Yes |
| 123 | CCDC15 |  |  | 197 | 0.419 | 0.4916 | Yes |
| 124 | MCM4 |  |  | 199 | 0.418 | 0.4933 | Yes |
| 125 | DSN1 |  |  | 202 | 0.413 | 0.4930 | Yes |
| 126 | SYNE2 |  |  | 203 | 0.413 | 0.4966 | Yes |
| 127 | TCF19 |  |  | 207 | 0.410 | 0.4943 | Yes |
| 128 | ZWINT |  |  | 212 | 0.407 | 0.4900 | Yes |
| 129 | LIG1 |  |  | 214 | 0.407 | 0.4916 | Yes |
| 130 | CCNE2 |  |  | 215 | 0.406 | 0.4952 | Yes |
| 131 | H2AFZ |  |  | 216 | 0.405 | 0.4987 | Yes |
| 132 | HSPB11 |  |  | 218 | 0.401 | 0.5003 | Yes |
| 133 | MNS1 |  |  | 219 | 0.401 | 0.5038 | Yes |
| 134 | CENPO |  |  | 223 | 0.398 | 0.5014 | Yes |
| 135 | FANCA |  |  | 227 | 0.395 | 0.4990 | Yes |
| 136 | TIMELESS |  |  | 228 | 0.395 | 0.5024 | Yes |
| 137 | STIL |  |  | 231 | 0.393 | 0.5020 | Yes |
| 138 | EZH2 |  |  | 236 | 0.388 | 0.4975 | Yes |
| 139 | VRK1 |  |  | 238 | 0.388 | 0.4990 | Yes |
| 140 | CDC6 |  |  | 239 | 0.388 | 0.5024 | Yes |
| 141 | PARPBP |  |  | 240 | 0.387 | 0.5058 | Yes |
| 142 | PCNA |  |  | 242 | 0.386 | 0.5072 | Yes |
| 143 | HMGN2 |  |  | 243 | 0.386 | 0.5106 | Yes |
| 144 | POLA1 |  |  | 247 | 0.383 | 0.5080 | Yes |
| 145 | RTKN2 |  |  | 248 | 0.379 | 0.5114 | Yes |
| 146 | CCNB2 |  |  | 250 | 0.379 | 0.5127 | Yes |
| 147 | JADE1 |  |  | 251 | 0.378 | 0.5160 | Yes |
| 148 | POLD3 |  |  | 252 | 0.377 | 0.5193 | Yes |
| 149 | MPHOSPH9 |  |  | 254 | 0.375 | 0.5206 | Yes |
| 150 | PLK4 |  |  | 255 | 0.374 | 0.5239 | Yes |
| 151 | RAD18 |  |  | 256 | 0.373 | 0.5272 | Yes |
| 152 | RFC5 |  |  | 260 | 0.370 | 0.5246 | Yes |
| 153 | CENPJ |  |  | 261 | 0.370 | 0.5278 | Yes |
| 154 | TUBB |  |  | 263 | 0.368 | 0.5290 | Yes |
| 155 | CENPW |  |  | 267 | 0.366 | 0.5264 | Yes |
| 156 | TUBG1 |  |  | 268 | 0.365 | 0.5296 | Yes |
| 157 | CEP57L1 |  |  | 269 | 0.364 | 0.5328 | Yes |
| 158 | SMC1A |  |  | 274 | 0.361 | 0.5281 | Yes |
| 159 | PCNT |  |  | 275 | 0.360 | 0.5312 | Yes |
| 160 | CHAF1A |  |  | 276 | 0.359 | 0.5344 | Yes |
| 161 | MMS22L |  |  | 277 | 0.359 | 0.5375 | Yes |
| 162 | ANP32E |  |  | 280 | 0.358 | 0.5367 | Yes |
| 163 | GINS1 |  |  | 283 | 0.357 | 0.5360 | Yes |
| 164 | PSIP1 |  |  | 284 | 0.357 | 0.5391 | Yes |
| 165 | CALM2 |  |  | 290 | 0.354 | 0.5324 | No |
| 166 | ZWILCH |  |  | 292 | 0.353 | 0.5335 | No |
| 167 | HAUS8 |  |  | 293 | 0.353 | 0.5366 | No |
| 168 | RFC4 |  |  | 295 | 0.351 | 0.5377 | No |
| 169 | SUZ12 |  |  | 297 | 0.349 | 0.5388 | No |
| 170 | DTL |  |  | 305 | 0.345 | 0.5281 | No |
| 171 | PRKDC |  |  | 306 | 0.344 | 0.5312 | No |
| 172 | CDC25C |  |  | 307 | 0.343 | 0.5342 | No |
| 173 | CENPN |  |  | 309 | 0.343 | 0.5352 | No |
| 174 | FAM111B |  |  | 314 | 0.342 | 0.5304 | No |
| 175 | DEK |  |  | 316 | 0.341 | 0.5314 | No |
| 176 | AKAP12 |  |  | 320 | 0.339 | 0.5285 | No |
| 177 | CSE1L |  |  | 321 | 0.338 | 0.5314 | No |
| 178 | RFC3 |  |  | 322 | 0.338 | 0.5344 | No |
| 179 | HAUS6 |  |  | 324 | 0.337 | 0.5354 | No |
| 180 | CIT |  |  | 326 | 0.336 | 0.5364 | No |
| 181 | RFC2 |  |  | 331 | 0.333 | 0.5315 | No |
| 182 | HIRIP3 |  |  | 336 | 0.331 | 0.5265 | No |
| 183 | BTG3 |  |  | 337 | 0.331 | 0.5294 | No |
| 184 | UBR7 |  |  | 340 | 0.330 | 0.5284 | No |
| 185 | NFATC2IP |  |  | 343 | 0.328 | 0.5274 | No |
| 186 | PHGDH |  |  | 344 | 0.328 | 0.5302 | No |
| 187 | HAT1 |  |  | 352 | 0.324 | 0.5194 | No |
| 188 | TMEM237 |  |  | 353 | 0.324 | 0.5222 | No |
| 189 | LMNB1 |  |  | 363 | 0.321 | 0.5074 | No |
| 190 | TOPBP1 |  |  | 369 | 0.319 | 0.5004 | No |
| 191 | PARP2 |  |  | 372 | 0.319 | 0.4993 | No |
| 192 | PRIM2 |  |  | 391 | 0.311 | 0.4668 | No |
| 193 | CEP192 |  |  | 395 | 0.310 | 0.4636 | No |
| 194 | BLM |  |  | 408 | 0.305 | 0.4428 | No |
| 195 | CENPC |  |  | 409 | 0.304 | 0.4455 | No |
| 196 | LRR1 |  |  | 410 | 0.304 | 0.4481 | No |
| 197 | PTTG1 |  |  | 413 | 0.302 | 0.4468 | No |
| 198 | ZGRF1 |  |  | 415 | 0.302 | 0.4475 | No |
| 199 | AHI1 |  |  | 425 | 0.299 | 0.4325 | No |
| 200 | SASS6 |  |  | 432 | 0.296 | 0.4234 | No |
| 201 | IQGAP3 |  |  | 447 | 0.294 | 0.3985 | No |
| 202 | TEX30 |  |  | 448 | 0.294 | 0.4011 | No |
| 203 | CCDC150 |  |  | 449 | 0.293 | 0.4037 | No |
| 204 | CBX5 |  |  | 457 | 0.291 | 0.3925 | No |
| 205 | SAE1 |  |  | 463 | 0.289 | 0.3853 | No |
| 206 | RFWD3 |  |  | 464 | 0.289 | 0.3878 | No |
| 207 | NUP155 |  |  | 468 | 0.288 | 0.3845 | No |
| 208 | NUP50 |  |  | 470 | 0.287 | 0.3850 | No |
| 209 | KCTD9 |  |  | 471 | 0.287 | 0.3875 | No |
| 210 | YEATS4 |  |  | 476 | 0.286 | 0.3822 | No |
| 211 | CHEK1 |  |  | 477 | 0.286 | 0.3847 | No |
| 212 | RNASEH2A |  |  | 478 | 0.285 | 0.3872 | No |
| 213 | NUCKS1 |  |  | 483 | 0.285 | 0.3819 | No |
| 214 | HADH |  |  | 485 | 0.284 | 0.3824 | No |
| 215 | HMGXB4 |  |  | 491 | 0.282 | 0.3751 | No |
| 216 | POLH |  |  | 492 | 0.282 | 0.3775 | No |
| 217 | CTDSPL2 |  |  | 495 | 0.282 | 0.3761 | No |
| 218 | PSMC3 |  |  | 506 | 0.280 | 0.3590 | No |
| 219 | WDHD1 |  |  | 513 | 0.278 | 0.3497 | No |
| 220 | TTF2 |  |  | 514 | 0.278 | 0.3521 | No |
| 221 | LSM3 |  |  | 522 | 0.277 | 0.3408 | No |
| 222 | DNMT1 |  |  | 523 | 0.277 | 0.3433 | No |
| 223 | ALYREF |  |  | 546 | 0.273 | 0.3026 | No |
| 224 | ACYP1 |  |  | 551 | 0.273 | 0.2971 | No |
| 225 | BRD8 |  |  | 562 | 0.270 | 0.2799 | No |
| 226 | GGH |  |  | 564 | 0.270 | 0.2803 | No |
| 227 | CDK2 |  |  | 565 | 0.270 | 0.2827 | No |
| 228 | DDX11 |  |  | 568 | 0.269 | 0.2811 | No |
| 229 | CTNNAL1 |  |  | 570 | 0.269 | 0.2815 | No |
| 230 | CNTRL |  |  | 575 | 0.269 | 0.2761 | No |
| 231 | PCDH19 |  |  | 581 | 0.268 | 0.2686 | No |
| 232 | RPA3 |  |  | 584 | 0.267 | 0.2671 | No |
| 233 | SLC43A3 |  |  | 590 | 0.265 | 0.2596 | No |
| 234 | OXCT1 |  |  | 614 | 0.261 | 0.2169 | No |
| 235 | CHCHD3 |  |  | 628 | 0.259 | 0.1937 | No |
| 236 | ARL6IP6 |  |  | 629 | 0.258 | 0.1959 | No |
| 237 | HNRNPUL1 |  |  | 637 | 0.257 | 0.1845 | No |
| 238 | EXOSC8 |  |  | 647 | 0.256 | 0.1691 | No |
| 239 | MAGI3 |  |  | 648 | 0.256 | 0.1714 | No |
| 240 | UHRF1 |  |  | 652 | 0.255 | 0.1677 | No |
| 241 | FAM76B |  |  | 653 | 0.255 | 0.1700 | No |
| 242 | RDX |  |  | 658 | 0.255 | 0.1644 | No |
| 243 | PCBP2 |  |  | 660 | 0.255 | 0.1646 | No |
| 244 | HNRNPUL2 |  |  | 672 | 0.252 | 0.1453 | No |
| 245 | SPATA5 |  |  | 675 | 0.252 | 0.1436 | No |
| 246 | MYBL1 |  |  | 678 | 0.251 | 0.1419 | No |
| 247 | CHD7 |  |  | 706 | -0.284 | 0.0915 | No |
| 248 | DBI |  |  | 740 | -0.422 | 0.0306 | No |
| 249 | HES6 |  |  | 752 | -0.523 | 0.0137 | No |
Table: GSEA details [plain text format]

  

Fig 2: RICHARDS\_NOWAKOWSKI\_SCIENCE\_2017\_MGE\_DIV\_UPREG: Random ES distribution      
 Gene set null distribution of ES for **RICHARDS\_NOWAKOWSKI\_SCIENCE\_2017\_MGE\_DIV\_UPREG**
